# A Heavy Metal and Trace Element Biomonitoring Study in a Young Cohort (Aged 18–24) in Istanbul, Turkey

**DOI:** 10.3390/ijerph23020233

**Published:** 2026-02-12

**Authors:** Nilay Topal, Meltem Pak Demir, Aydanur Kulaç, Bulut Yurtsever, Demet Dinç, Fehime Aksungar

**Affiliations:** 1 Biochemistry and Molecular Biology Department, Graduate School of Health Sciences, Acıbadem Mehmet AliAydınlar University, 34752 Istanbul, Turkey; 2 Department of Public Health, School of Medicine, Acıbadem Mehmet Ali Aydınlar University, 34752 Istanbul, Turkey; 3 Department of Biochemistry, School of Medicine, Acıbadem Mehmet Ali Aydınlar University, 34752 Istanbul, Turkey

**Keywords:** heavy metal, trace element, ICP-MS, arsenic, mercury, young adult

## Abstract

This study aimed to determine the exposure levels of young individuals living in Istanbul, a region in Turkey with a high population density and significant environmental pollution, by measuring the levels of heavy metals and trace elements in blood, serum, and urine. A total of 95 young people aged 18–24 participated in the study. Toxic heavy metals (Pb, As, Hg, Cd, and Cr) and physiological trace elements (Cu, Zn, Se, Mo, Mn, and Co) were measured in participants’ whole blood, serum, and urine samples using the ICP-MS technique. Participants were stratified by gender, as differences in body surface area may affect the absorption and metabolism of trace elements, and by smoking status, since smoking is a recognized source of heavy metal exposure. Gender differences revealed that blood lead levels were higher in males (*p* < 0.05), while manganese levels were higher in females (*p* < 0.05). When serum samples were analyzed, males had significantly higher zinc (*p* < 0.05) and selenium (*p* < 0.05) levels compared to females, whereas females had significantly higher levels of copper (*p* < 0.05) and cobalt (*p* < 0.05). Similar differences for copper (*p* < 0.05) and cobalt (*p* < 0.05) were observed in urine samples, with higher levels found in females. Blood cadmium levels were found to be significantly higher in smokers (*p* < 0.05). This biomonitoring study is one of the rare studies conducted in this region to assess heavy metal exposure among young adults.

## 1. Introduction

Heavy metals are defined as metals with a density greater than 5 g/cm^3^ [1]. Naturally occurring in forms of hydroxides, oxides, sulfates, silicates, and organic compounds, heavy metals are dispersed into the environment through various natural processes [2]. However, the increased use of heavy metals in industrial, domestic, medical, agricultural, and technological applications over the last century has significantly elevated their interaction with living organisms [3]. 

Industries most responsible for the dissemination of heavy metals into the ecosphere include the iron and steel industry, waste incineration and energy production facilities, fertilizer production, chlor-alkali manufacturing, and glass production [4,5]. Heavy metals originating from industrial wastewater are largely transferred to and concentrated in sewage sludge during wastewater treatment while the dissolved fractions reach surface and marine waters [6,7]. From these sources, heavy metals infiltrate into drinking water, air, and soil, eventually entering the food chain [8]. As heavy metals accumulate through the food chain, they are mainly introduced into the human body via ingestion, while inhalation and dermal contact represent additional exposure routes in contaminated environments [9]. Heavy metals such as cadmium, lead, and mercury have long biological half-lives (ranging from years to decades) due to their strong binding to tissue proteins and inefficient metabolic excretion, leading to cumulative accumulation in organs [1,9,10,11]. Heavy metal exposure during early childhood should be considered in the differential diagnosis of individuals who later develop neurological or systemic manifestations. However, metal concentrations are often within normal ranges by that time, which complicates the confirmation of past toxicity [12]. 

Given that heavy metal accumulation often begins early in life, assessing body burdens across different age groups is essential. Monitoring heavy metal levels in young people is particularly important, as it establishes baseline values for future reference and enables longitudinal tracking of exposure and potential health effects throughout life. Moreover, young adulthood (18–24 years) represents a critical transitional life stage for environmental biomonitoring, as individuals have typically completed childhood exposure pathways while having minimal cumulative occupational exposure. This age group has therefore been increasingly used to establish baseline heavy metal exposure levels prior to long-term accumulation and age-related metabolic variability [13,14]. Despite its relevance, biomonitoring data focusing specifically on young adults remain limited in Turkey, creating an important gap in age-stratified exposure assessment.

Istanbul, Turkey’s most industrialized and polluted metropolitan area, presents heightened exposure risks due to traffic emissions, industrial activities, maritime transport, and construction-related particulate matter. This study aims to quantify selected heavy metals in the blood, serum, and urine of young people in Istanbul, generating biomonitoring data to characterize environmental exposure patterns and support public health risk assessments. The selection of heavy metals and trace elements was guided by their toxicological and physiological relevance. We included toxic heavy metals (Pb, As, Hg, Cd, and Cr) because they are among the most commonly recognized environmental contaminants, with well-documented adverse health effects such as neurotoxicity, nephrotoxicity, carcinogenicity, and cardiovascular risk, even at low exposure levels [1,4,9,10,11].

## 2. Material and Methods

### 2.1. Study Area

**Figure 1:**
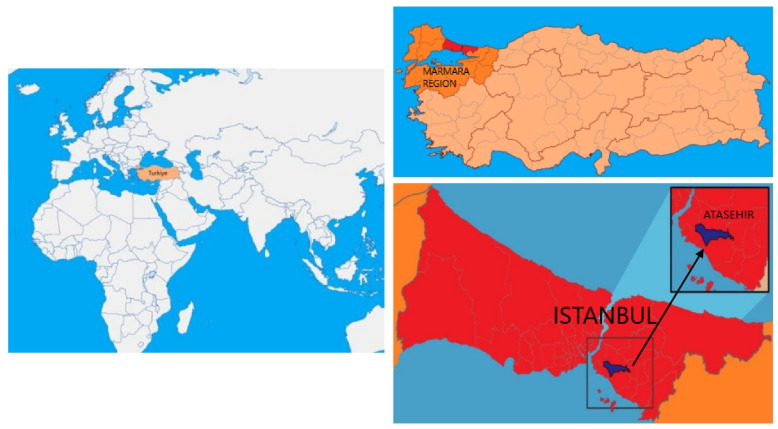
Sample collection region.

Istanbul, located in the northwest of Turkey’s Marmara Region, has a population of approximately 16 million, making it the most populous city in Turkey. Sample collection was carried out in the Ataşehir district (Figure 1). Ataşehir is a densely populated district in Istanbul, marked by heavy traffic, nearby industrial activity, and rapid urban development. These factors contribute to potential environmental pollution, including heavy metals. Its population density and urban characteristics make it a relevant area for assessing environmental exposure in young individuals. The young participants comprise university students who will be subjected to periodic biomonitoring throughout the course of their academic tenure. This longitudinal approach will facilitate the assessment of temporal changes in exposure and enable a more comprehensive evaluation of potential cumulative effects.

### 2.2. Sample Collection

A total of 95 participants, including 56 males and 39 females aged between 18 and 24, voluntarily participated in our study. All participants were students of Acıbadem Mehmet Ali Aydınlar University, located in Ataşehir district. The study was approved by the Ethics Committee of Acıbadem Mehmet Ali Aydınlar University with the decision dated 25 November 2022, numbered 2022-18/12. Prior to the study, initial interviews were conducted with the participants to ensure they met the inclusion criteria. Following a 10–12 h fasting period, venous blood was collected in 3 mL K_2_EDTA tubes for whole blood analysis, serum was obtained in 6 mL trace element tubes containing a clot activator, and urine samples were collected in sterile urine containers. We required a 10–12 h overnight fasting period prior to sample collection in order to minimize the potential influence of recent dietary intake and circadian variations of blood and urine concentrations of metals and trace elements. All the samples were collected within three days, and participants signed the informed consent forms while receiving verbal information about the study. Immediately after sample collection, participants completed a detailed 24-question survey, including socio-economic, dietary, health, and demographic information (Supplementary S1). The questionnaire included qualitative dietary variables such as frequency of seafood consumption, red meat intake, fruit and vegetable consumption, and use of dietary supplements. These variables were analyzed descriptively and were not used to perform quantitative exposure estimation or to model dietary intake of specific trace elements. Trace element status, including selenium, copper, mercury, and arsenic, is known to be strongly influenced by dietary patterns. Dietary items in the questionnaire allowed only qualitative characterization of dietary habits and precluded estimation of nutrient or trace element intake.

### 2.3. Sample Preparation

Blood samples collected in trace element tubes (trace element tubes are specially designed for the accurate measurement of elements present in very low concentrations, and are manufactured and processed in metal-free environments to prevent external contamination obtained from BD Vacutainer^®^, Franklin Lakes, NJ, USA) were centrifuged at 3500 rpm for 10 min, and the separated serums were transferred into newly labeled trace element tubes. Urine samples were also transferred from sterile urine containers to numbered sterile 15 mL Falcon tubes used for trace element measurements. Whole blood, serum, and urine samples were transferred to the laboratory in foam boxes with ice packs. The samples were stored at 2–8 °C until the working hours and were processed within a maximum of 24 h.

### 2.4. Heavy Metal and Trace Element Measurements

All metal measurements were performed using an Agilent Technologies 7500c ICP-MS device. The internal standard mix and tuning solution were obtained from Agilent Technologies (Santa Clara, CA, USA), while nitric acid, Triton X-100, butanol, and the standards for each element were obtained from Sigma Aldrich (Saint Louis, MO, USA). 0.5 g of EDTA was weighed using a precision balance and transferred into a 500 mL Triton X-100 volumetric flask. After adding 25 mL of butanol and 22 mL of ammonia to the flask, the final volume was adjusted to 1000 mL with distilled water to prepare the Triton solution. 5.4 mL of 65% nitric acid was added to a separate 500 mL volumetric flask, and the final volume was adjusted to 500 mL with distilled water to prepare a 1% nitric acid solution. Intermediate stock solutions of 50 µg/mL (ppm) were prepared for copper and zinc elements. To do this, 250 µL of each element’s 10,000 µg/mL stock solution was pipetted and transferred into 50 mL volumetric flasks containing Triton. Intermediate stock solutions of 100 µg/mL (ppm) were prepared for the other elements. To do this, 500 µL of each element’s 10,000 µg/mL stock solution was pipetted and transferred into 50 mL volumetric flasks containing Triton. Mix solutions for serum, whole blood, and urine were prepared as follows. In 50 mL volumetric flasks, the following volumes of the intermediate stock solutions for each element were added: 2500 µL of Pb, 100 µL of As, 250 µL of Hg, 100 µL of Cd, 25 µL of Cr, 250 µL of Se, 25 µL of Mo, 100 µL of Mn, 50 µL of Co, 5000 µL of Cu, and 5000 µL of Zn. Finally, the volume of each solution was adjusted to 50 mL with Triton, and these stock solutions were labeled as Standard-9. A standard series from Standard-9 to Standard-1 was prepared through serial dilution. The LOD (Limit of Detection) and LOQ (Limit of Quantification) values for whole blood, serum, and urine measurements are provided in Table 1. The analytical method was fully validated according to international guidelines, and recovery, accuracy, and precision parameters for As and Pb were found to be within acceptable ranges [15,16]. In addition, interference correction protocols were applied during ICP-MS analysis to mitigate potential polyatomic interferences, and appropriate internal standards were used to correct for matrix effects and instrument drift [16]. Therefore, while a unified protocol was used, its suitability for each analyte was confirmed during method validation [15,16].

The limits of detection (LOD) and quantification (LOQ) were determined in accordance with international bioanalytical method validation guidelines [16]. Briefly, LOD was calculated as three times the standard deviation (SD) of replicate measurements of a low-concentration spiked sample divided by the slope (S) of the calibration curve (LOD = 3 × SD/S), while LOQ was calculated as ten times the SD divided by the slope (LOQ = 10 × SD/S). These calculations were based on at least six replicate measurements near the expected lower limit of quantification. This approach is widely accepted for trace-level quantification in biological matrices, including heavy metal analysis by ICP-MS [16].

In the present study, quantitative analysis of trace and heavy metals (Pb, As, Hg, Cd, Cr, Cu, Zn, Se, Mo, Mn, and Co) was performed using inductively coupled plasma mass spectrometry (ICP-MS). The instrument was operated in standard mode, and the scanned mass-to-charge (*m*/*z*) range extended from 7 to 240, which encompasses the target analytes as well as potential interfering species [16]. Below are the monitored isotopes (*m*/*z* values) for each element:

- Lead (Pb): *m*/*z* 206, 207, 208
- Arsenic (As): *m*/*z* 75
- Mercury (Hg): *m*/*z* 200, 202
- Cadmium (Cd): *m*/*z* 111, 114
- Chromium (Cr): *m*/*z* 52
- Copper (Cu): *m*/*z* 63, 65
- Zinc (Zn): *m*/*z* 64, 66, 68
- Selenium (Se): m/z 78, 82
- Molybdenum (Mo): *m*/*z* 95, 98
- Manganese (Mn): *m*/*z* 55
- Cobalt (Co): *m*/*z* 59

High-purity argon gas (purity ≥ 99.999%) was used as the plasma, auxiliary, and nebulizer gas throughout the ICP-MS analysis. The use of high-grade argon minimizes polyatomic interferences and ensures stable plasma conditions, thereby enhancing analytical accuracy and precision [16]. 

To ensure analytical accuracy and precision, two concentration levels (Level I and Level II) of RECIPE ClinChek^®^ Whole Blood Control, Urine Control, and Serum Control materials (RECIPE Chemicals + Instruments GmbH, Munich, Germany) were employed for each biological matrix. These are commercially available lyophilized certified reference materials specifically designed for trace and toxic element monitoring in clinical and environmental studies [15]. Each control level contains assigned target concentrations for a broad panel of elements, including those measured in our study (e.g., Pb, As, Hg, Cd, etc.), and is traceable to international reference standards [16]. The controls were analyzed in parallel with study samples within each analytical run to monitor intra-run performance characteristics. The use of dual-level controls allowed us to assess both the within-run precision (repeatability) and analytical accuracy across low and high concentration ranges, as recommended by international bioanalytical validation guidelines [15]. The samples were then ready for analysis. Measuring all matrices allows for a more comprehensive assessment of metal toxicokinetics, transport, and biological availability, and facilitates comparison with existing studies that report results in different biological matrices. While working with whole blood samples, the device was washed with Triton between each sample. When working with urine samples, the device was washed with 1% nitric acid after every 10 samples. The operating conditions for the ICP-MS device used for all metal measurements were as follows: RF power 1500 W, nebulizer gas flow rate 0.75 L/min, make-up gas flow rate 0.19 L/min, sample uptake 0.7 mL/min, sampling depth 5 mm, sampler 1.0 mm Pt, skimmer 0.4 mm Pt, and dwell time/isotope 500 ms. Measurements were performed in triplicate.

### 2.5. Statistical Analysis

The data were compared using parametric and non-parametric methods. A significance level of alpha = 0.95 was considered. Data were analyzed using one-way analysis of variance (ANOVA) to compare differences among groups. When a significant overall effect was detected, post hoc comparisons were performed using the least significant difference (LSD) test. Results are presented as mean ± standard deviation (SD). A *p* value of <0.05 was considered statistically significant. All statistical analyses were conducted using GraphPad Prism 9.5.1 software. Differences between smoking and gender were tested with normal standard deviation tests separately for each matrix.

## 3. Results

After the initial participant recruitment and survey evaluation, strict inclusion and exclusion criteria were applied to ensure data quality and reliability. Participants were included if they were within the target age range (18–24 years), were residents of the specified study region, and had provided complete blood, serum, and urine samples. Exclusion criteria encompassed individuals diagnosed with chronic diseases (e.g., diabetes, cardiovascular diseases), those regularly using medications that could potentially affect heavy metal metabolism or biomarker levels, and participants whose biological samples were incomplete, or insufficient for analysis. This approach was designed to minimize confounding factors that might influence heavy metal levels and to ensure that the study population accurately represents healthy young adults exposed to environmental conditions. Of the 95 initially recruited individuals, 12 were excluded based on predefined criteria: 5 participants due to the presence of chronic diseases, 4 due to regular use of medications potentially affecting heavy metal metabolism, and 3 due to incomplete biological samples. Consequently, the final analysis was conducted on data from 83 volunteers.

Based on the responses from the participants, the demographic characteristics of the study group were recorded (Table 2). A total of 29 females and 54 males were included in the study, and when comparing the participants’ age, body mass index, and body surface area, it was found that the only significant difference was in the body surface area in males, indicating a homogeneous community (Table 2). Participants were stratified by gender, given that variations in body surface area can influence the absorption, distribution, and metabolism of trace elements, potentially affecting heavy metal levels. Additionally, since smoking constitutes a known source of exposure to certain heavy metals, participants were further categorized based on their smoking status. This stratification enabled a more precise differentiation between environmental and smoking-related exposures (Table 3).

**Figure 2:**
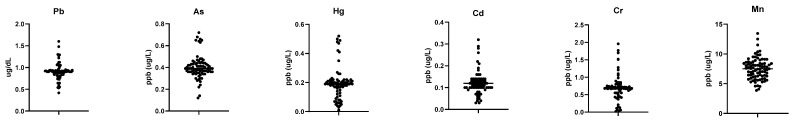
Distribution graphs of heavy metals analyzed in blood samples.

**Figure 3:**
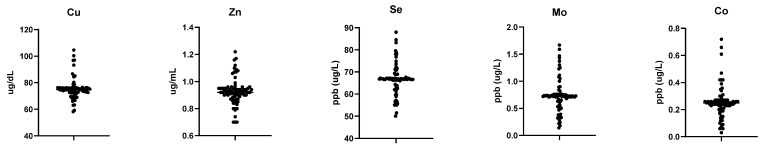
Distribution graphs of heavy metals analyzed in serum samples.

**Figure 4:**
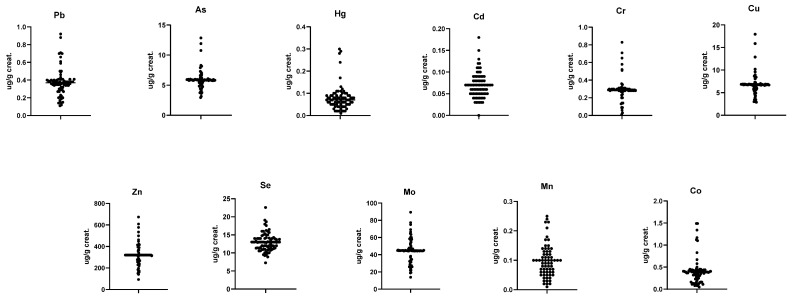
Distribution graphs of heavy metals analyzed in urine samples.

The mean ± SD values of heavy metals and trace elements measured from blood, serum, and urine samples are presented in Table 4 and Table 5, while the distribution graphs are shown in Figure 2, Figure 3 and Figure 4. Smoking was associated with significantly higher cadmium concentrations in both blood and urine, whereas no statistically significant differences were observed for the other heavy metals.

## 4. Discussion

Industrial use of heavy metals has substantially increased human exposure in recent decades, with adverse health effects now recognized as more severe than previously assumed. Neurological manifestations commonly attributed to stress or fatigue may, in fact, result from bioaccumulation of certain heavy metals, particularly when exposure begins in early life. According to the 2022 WHO Ambient Air Quality Database and reports from the Turkish Ministry of Environment and Urbanization, İstanbul frequently exceeds recommended thresholds for particulate matter (PM2.5 and PM10) and nitrogen dioxide (NO_2_), reflecting substantial levels of air pollution with well-documented adverse health implications [17,18]. Within this environmental context, the evaluation of toxic heavy metal exposure in young adults is of particular importance, as early-life accumulation of these elements may initiate long-term biological damage. Chronic exposure, even at subclinical levels, has been associated with an increased risk of neurodegenerative disorders, impaired cognitive function, and progressive organ injury over the life course [1,2,9].

The primary mechanism of heavy metal toxicity involves the proliferation of reactive oxygen species (ROS), causing oxidative damage in the body. Additionally, heavy metals bind to specific functional groups of proteins, such as sulfhydryl (–SH) and amino groups, leading to functional impairment and the accumulation of pathological proteins in certain tissues. These mechanisms cause cells, particularly neurons, to undergo apoptosis, resulting in chronic neuronal loss and various neurological symptoms. Moreover, some heavy metals compete with and replace metals with physiological functions. For instance, lead mimics calcium pathways, accumulating in neurons as well as in bones and gingiva. Similarly, the body treats thallium like potassium and arsenic like phosphorus [4,19,20,21]. While heavy metals primarily interact with specific target molecules (e.g., proteins, enzymes) or cells, the resulting cellular damage can lead to dysfunction in multiple organs and systems sensitive to these metals [4,22]. These effects include organ dysfunction, metabolic abnormalities, hormonal disruptions, congenital disorders, immune system impairments, and cancer [4,23,24,25]. Neurological findings associated with heavy metals span a broad spectrum and are included in the differential diagnosis of many diseases. For example, heavy metal exposure should be considered in cases of benign positional vertigo, memory loss, essential tremor, Alzheimer’s disease, multiple sclerosis, and dementia. Furthermore, exposure to these metals at any stage of life does not preclude the delayed manifestation of related clinical or subclinical effects [26]. Heavy metals, particularly those encountered during childhood, can accumulate in the body and lead to severe diseases over time. In the present study, we aimed to determine the burden of heavy metals among young individuals residing in İstanbul, and to compare the concentrations measured in blood, serum, and urine with reference data from other regions of Turkey and international cohorts. Such comparative analysis provides critical insights into the potential future public health burden associated with environmental toxicant exposure in metropolitan settings. In addition to toxic heavy metals, this study also evaluated physiological trace elements, since essential trace elements such as zinc, copper, and selenium play critical roles in antioxidant defense, enzymatic regulation, and neurocognitive development. Alterations in their homeostasis may not only impair normal physiological functions but also modulate the toxicity of heavy metals by influencing absorption, distribution, and detoxification pathways [27].

**Figure 5:**
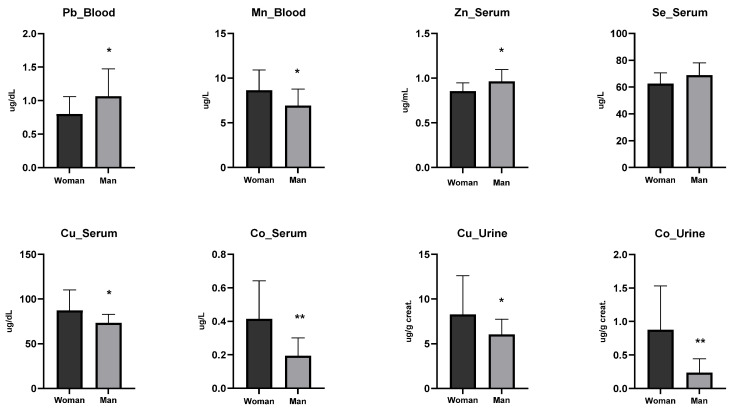
Comparison of heavy metal concentrations stratified by smoking status. Note: * *p* < 0.05, ** *p* < 0.0001.

In our study, the overall concentrations of toxic heavy metals except blood chromium levels were found to be below the MAL values reported in the NHANES data (Table 4), indicating that, while exposure at this age did not exceed the safe thresholds, documenting these concentrations is important for future assessments as the population ages. Briefly, assessment of physiological trace elements revealed that selenium and copper concentrations were lower than the expected reference ranges, whereas other trace elements remained within their respective normal limits (Table 5). Additionally, participants were stratified by gender, recognizing that differences in body surface area may influence the absorption and metabolism of trace elements, and by smoking status, given that tobacco use is a known source of heavy metal exposure. Gender-specific analyses demonstrated that blood lead levels were significantly higher in males, while manganese levels were elevated in females (Figure 5). Upon analysis of serum samples, it was shown that males had significantly higher concentrations of zinc (Figure 5), whereas females exhibited significantly higher levels of copper and cobalt (Figure 5). Although there was no statistical difference in selenium, the averages were found to be higher in men. Meanwhile, comparable gender-related differences in copper and cobalt were observed in urine samples, with females consistently showing higher concentrations (Figure 5). The observed low levels of selenium and copper in the study cohort may be attributed to several factors. Dietary intake represents the primary source of these essential trace elements, and insufficient consumption of selenium and copper-rich foods could contribute to suboptimal status. Regional variations in soil content and agricultural practices may also influence the elemental composition of locally produced foods, particularly for selenium, which is highly dependent on soil concentration [28]. Additionally, lifestyle factors such as smoking, alcohol consumption, and physical activity can affect trace element absorption and metabolism. Gender-related physiological differences, including variations in hormonal regulation and body composition, may further modulate circulating levels of these elements [29]. While the detected concentrations remained within subclinical ranges, these findings highlight the need for monitoring essential trace elements to prevent potential long-term health implications.

For comparison with previous studies, the concentrations of heavy metals and trace elements obtained in this study were compared with values reported in previous studies from Turkey and other countries with similar age groups. Table 6 presents these comparisons, highlighting similarities and differences in exposure levels across populations.

When considering each heavy metal and trace element on its own, different trends were observed: In developed countries, the use of lead in household items and paints has been banned since the 1970s, and its use in gasoline has been prohibited since the 1980s. Numerous studies on this subject have shown that lead exposure has declined significantly due to these bans [43]. Earlier studies in Turkey reported higher blood lead levels among participants compared to our study (Table 6). Over time, a decline in lead exposure has been observed, likely due to environmental regulations such as the ban on leaded gasoline, which contributed to lower blood lead levels in recent years. The complete ban on the use of leaded gasoline in our country in 2004 has contributed to the observed decline in lead exposure over two decades. The U.S. Centers for Disease Control and Prevention (CDC) set a critical threshold of 10 µg/dL for blood lead levels, but continues to advocate for levels below 1 µg/dL for all age groups, particularly for children [44,45,46,47]. The average blood lead levels in our study (Table 4) were found to be below this threshold. Similarly, the average urinary lead levels in our study were lower than the average urinary lead levels of adults aged 20 and older reported in the NHANES data covering the 2011–2018 period (Table 4). The higher blood and urinary lead levels reported in these previous studies compared to our findings are likely attributable to the inclusion of older adult age groups, who typically exhibit greater age-related lead accumulation. These observations underscore the importance of longitudinal monitoring of lead exposure as individuals age. Accordingly, a follow-up of the participants in this study is planned at a two-year interval to assess changes in lead burden over time.

Regarding arsenic levels, differences were observed between our findings and those reported in previous international studies (Table 6). Blood and urinary arsenic levels in our study were lower compared to some European studies and NHANES. Along with this, our urinary arsenic levels were comparable to Canadian Health Measures Survey (CHMS) data (Table 4 and Table 6). Arsenic levels can vary, which is attributed to regional environmental factors and dietary habits, particularly the consumption of drinking water and seafood [48,49]. 

Many international organizations have established MAL values for blood and urinary mercury levels in healthy individuals [50,51]. Although thresholds vary slightly between guidelines, our study found both blood and urinary mercury levels to be well below these accepted limits (Table 4). Compared with previous international and national reports, including data from the U.S., Canada, France, and Turkey, the mercury levels observed in our young participants were consistently lower, reflecting minimal exposure in this population (Table 4 and Table 6). This low mercury exposure in our study population may be attributed to several factors. Reduced consumption of high-mercury foods, such as certain fish and seafood, along with improved environmental regulations and monitoring of industrial emissions, likely contribute to the lower levels observed. Additionally, regional differences in dietary habits, water sources, and overall environmental contamination can further explain the variation in mercury levels across populations. These findings highlight the importance of continuous monitoring to assess population-level exposure and the effectiveness of public health interventions.

For individuals without occupational exposure, the primary sources of cadmium exposure are diet and tobacco products. Beyond these, industrial incidents constitute the most significant risk factor [52,53]. According to the International Agency for Research on Cancer (IARC), the acceptable blood cadmium concentration levels for healthy individuals who are non-smokers and smokers, without environmental or occupational cadmium exposure, are reported as 0.4–1 μg/L and 1.4–4 μg/L, respectively [54]. However, under conditions of environmental exposure, these levels may exceed 10 μg/L [54] Previous studies have reported varying blood and urinary cadmium levels across different populations and age groups (Table 6). In our study, although there was a clear difference in cadmium levels between smokers and non-smokers, both blood and urinary cadmium concentrations were well below the maximum allowable limit values (MAL values) established by international guidelines and lower than those reported in studies conducted in Turkey, Canada, the U.S., and France (Table 4 and Table 6).

In our study, the mean blood chromium level in young individuals exceeded the normal human MAL reported by the NHANES (Table 4) and was higher than the values reported for the Canadian population (Table 6), indicating relatively elevated exposure in this cohort. The general population is exposed to chromium through the inhalation of ambient air, food and water intake, and the use of fossil fuels, tobacco products, and industrial products [55]. As a result of the use of fossil fuels in industry, transportation, and heating, the concentration of chromium in urban air is 30 times higher than in rural areas. Due to smoking, indoor air contaminated with chromium can contain up to 400 times more chromium than outdoor air [56]. Additionally, chromium chemicals are used in various fields such as dental implants, water treatment, ceramics, canned food containers, and polishing materials [56]. The high chromium levels found in our study may be related to environmental exposure, possibly due to young individuals spending significant time in indoor social environments contaminated with tobacco products (e.g., hookah, cigarettes), living in high-pollution areas, and having high consumption of fast food. Some types of food packaging coatings can release trace amounts of chromium into food, contributing to overall exposure. However, the present study focused on biological monitoring and did not include concurrent measurements of environmental media such as air, drinking water, food, or soil. Biomonitoring reflects integrated internal exposure from multiple pathways, including dietary intake, inhalation, and dermal absorption, rather than direct source-specific exposure. Consequently, the results should be interpreted as indicators of overall body burden rather than evidence of particular environmental contamination sources. Future studies would benefit from integrated environmental co-monitoring approaches combining biological measurements with parallel assessment of relevant environmental matrices to enable more robust exposure source identification and risk assessment.

Regional studies in Turkey reported average serum copper levels of 137.8 µg/dL in Tokat [38] and 138 ± 14 µg/dL in Denizli [39], while a French study reported 107 µg/dL [36] (Table 6). Our participants had markedly lower levels, though still within the normal adult range (Table 5). Low copper is often linked to excessive iron or zinc intake or insufficient dietary copper, and major sources include organ meats, fish, nuts, grains, and green vegetables [53]. These findings suggest that dietary habits may contribute to the reduced levels observed. Besides dietary factors, several mechanisms may also contribute to the lower serum copper levels observed in our study group. Copper homeostasis is influenced by gastrointestinal absorption and hepatic metabolism, and variations in these processes can result in reduced circulating levels. Genetic polymorphisms affecting copper transport proteins (e.g., ATP7A, ATP7B) may also play a role [57,58]. These factors, alone or in combination, could partly explain the lower copper levels detected in our participants.

The serum selenium levels observed in our study group were lower than the reference values established for healthy individuals (Table 5), and also, similarly, our values were lower than the reference values reported for healthy individuals in France [36] (Table 6). Consistent with our findings, previous studies conducted in different regions of Turkey [38,40,41,42] have also reported lower selenium concentrations compared with the reference data, suggesting that selenium insufficiency may be a widespread issue in Turkey (Table 6). Selenium deficiency often arises from low soil content and insufficient dietary intake. Previous studies have reported relatively low selenium content in certain agricultural soils in Turkey, which may influence food selenium levels; however, individual exposure is determined by multiple factors, including dietary diversity and food sourcing [59]. 

Functionally, reduced selenium impairs the activity of selenoproteins such as Glutathione Peroxidase (GPx), which play a key role in antioxidant defense and neuroprotection [60]. Experimental and clinical studies have demonstrated that inadequate GPx activity contributes to neuronal vulnerability, oxidative stress, and pathological processes resembling those seen in neurodegenerative diseases such as Parkinson’s and Alzheimer’s [61]. Moreover, selenium supplementation has been associated with improved cognitive performance in Down syndrome, as well as reduced relapse rates in multiple sclerosis when combined with vitamin E [62,63]. Selenium deficiency has also been linked to mood disturbances, behavioral changes, and altered neurotransmitter turnover [64]. Taken together, these findings suggest that the lower selenium levels detected in our cohort may have significant implications for both antioxidant capacity and neurological health.

When heavy metal levels were compared by gender (Figure 5), men showed significantly higher blood lead concentrations (*p* = 0.028). Lead accumulates in erythrocytes, and the higher hematocrit in men may increase retention, while menstrual blood loss contributes to lower levels in women [65]. In contrast, blood manganese levels were higher in women (*p* = 0.001), consistent with studies showing sex- and age-related variations. Manganese rises during pregnancy and is influenced by estrogen, while iron deficiency, more common in women, enhances manganese absorption [66,67,68]. Serum zinc (*p* = 0.0058) and selenium (*p* = 0.0272) were higher in men, likely due to testosterone-driven differences in mineral metabolism, greater muscle mass, and enhanced utilization in antioxidant enzymes [69,70,71]. By contrast, women had higher serum and urinary copper and cobalt levels, associated with estrogen-mediated regulation of ceruloplasmin and trace element metabolism, as well as differences in body composition and energy metabolism [72,73].

This study has some inherent limitations that should be considered when interpreting the findings. The relatively small sample size, while adequate to represent the target age group, may restrict the generalizability of the findings. The study population was drawn exclusively from participants residing in Istanbul and the Marmara region, which may not fully reflect exposure patterns in other areas of Turkey. Additionally, the cross-sectional design captures data at a single time point, limiting the evaluation of long-term exposure trends. Although comprehensive information on lifestyle factors, diet, alcohol consumption, seafood intake, and medication use was obtained through questionnaires, unreported or unrecognized differences among participants may still have influenced the results. Moreover, the study was conducted in a university-based sample of young adults, which represents a relatively homogeneous subgroup in terms of educational background and age. Such populations may differ from the general young adult population with respect to socioeconomic status, lifestyle factors, and environmental exposure patterns. The age range of 18–24 years was selected to represent young adulthood, a life stage characterized by the relative stabilization of physiological systems and the absence of age-related chronic disease, while still reflecting cumulative environmental exposure during childhood and adolescence. Focusing on this age group allows for the assessment of baseline exposure levels prior to long-term occupational or disease-related confounding. However, due to the specific characteristics of the study population, the findings should be interpreted with caution and should not be generalized. Larger population-based studies incorporating diverse socioeconomic and demographic groups are needed to confirm and extend these observations. Nevertheless, in spite of these limitations, the study provides valuable insight into heavy metal exposure in young individuals and lays the groundwork for future longitudinal investigations. Monitoring heavy metal levels across age groups has become an integral component of public health strategies in many countries [13,14,74]. In Turkey, however, available data remain limited to regional studies, and overall awareness is still insufficient. The present study contributes to addressing this gap by evaluating heavy metal concentrations in blood, serum, and urine samples of young individuals residing in Istanbul, one of the most polluted regions in the country. Despite its modest sample size, the study adequately represents this cohort and, to our knowledge, is the first in Istanbul and the Marmara region to examine heavy metal distribution in young individuals.

Reference biomonitoring data from rapidly urbanizing regions remain limited, and such data are essential for international comparisons, exposure trend analysis, and risk assessment frameworks. Therefore, we believe that the findings are relevant not only for local authorities but also for the global scientific community engaged in environmental health and exposure science. Moreover, the inclusion of university students, who can be followed prospectively with repeated measurements, provides a unique opportunity for longitudinal evaluation. Importantly, these data also contribute to the limited international evidence on heavy metal exposure in young people, an age group for which global information remains scarce, thereby enhancing the broader understanding of exposure patterns worldwide.

In conclusion, this study provides important baseline data on heavy metal exposure in young individuals by evaluating concentrations in blood, serum, and urine samples. The findings highlight measurable exposure levels in a population residing in Istanbul and the Marmara region, one of the most industrialized and polluted areas of Turkey. These findings underscore the need for larger, multicenter, and longitudinal investigations across different regions of Turkey to better characterize temporal trends, regional differences, and potential health impacts of chronic heavy metal exposure. Such efforts would support the development of evidence-based public health policies and targeted prevention strategies.

## Figures and Tables

**Table 1:** LOD and LOQ values.

Metals	LOD			LOQ		
	Blood	Serum	Urine µg/g Creat	Blood	Serum	Urine µg/g Creat
Pb_µg/dL	0.012	0.015	0.030	0.025	0.025	0.050
As_ppb (µg/L)	0.040	0.025	0.025	0.050	0.050	0.050
Hg_ppb (µg/L)	0.010	0.010	0.010	0.025	0.025	0.025
Cd_ppb (µg/L)	0.010	0.010	0.010	0.025	0.025	0.025
Cr_ppb (µg/L)	0.018	0.025	0.012	0.025	0.040	0.020
Cu_µg/dL	0.700	0.700	0.050	1.500	1.500	0.075
Zn_µg/mL	0.010	0.012	0.015	0.020	0.020	0.025
Se_ppb (µg/L)	0.600	0.500	0.050	1.200	1.800	0.100
Mo_ppb (µg/L)	0.020	0.018	0.020	0.040	0.050	0.050
Mn_ppb (µg/L)	0.050	0.050	0.010	0.075	0.075	0.015
Co_ppb (µg/L)	0.020	0.030	0.030	0.025	0.050	0.050

Note: LOD: Limit of Detection, LOQ: Limit of Quantification; creat: creatinine.

**Table 2:** Demographic characteristics of the participants.

	Woman	Man
n	29	54
Age (year)	21.0 ± 2.0	20.5 ± 1.8
Weight (kg)	66.3 ± 14.1	78.5 ± 11.4
Height (cm)	1.6 ± 0.06	1.7 ± 0.06
BMI (kg/m^2^)	24.5 ± 5.4	24.5 ± 2.9
BSA (m^2^)	1.5 ± 0.3	1.9 ± 0.3

Values are presented as mean ± SD. BSA values were calculated using the DuBois formula (BMI = Body Mass Index, BSA = Body Surface Area). Independent *t*-tests showed expected sex-related differences in weight and height between males and females.

**Table 3:** Gender-based distribution of smoking status among study participants.

	Woman		Man	
	*n* = 29	%	*n* = 54	%
Smoking	10	34	17	31
Non-Smoking	19	66	37	69

**Table 4:** Mean values of heavy metals in blood and urine.

Heavy Metals	Matrix	This Study (Mean ± SD)	Maximum Allowable Limit (MAL) *
Pb (µg/dL)	Whole blood	0.91 ± 0.18	2.62
Pb (µg/g creat)	Urine	0.37 ± 0.15	1.09
As (µg/L)	Whole blood	0.40 ± 0.10	1.0 **
As (µg/g creat)	Urine	5.90 ± 1.53	59.7
Hg (µg/L)	Whole blood	0.19 ± 0.11	4.36
Hg (µg/g creat)	Urine	0.08 ± 0.05	1.0
Cd (µg/L)	Whole blood	0.12 ± 0.05	1.44
Cd (µg/g creat)	Urine	0.07 ± 0.03	0.91
Cr (µg/L)	Whole blood	0.71 ± 0.37	0.62
Cr (µg/g creat)	Urine	0.30 ± 0.13	1.09
Mn (µg/L)	Whole blood	7.51 ± 1.79	16.0
Mn (µg/g creat)	Urine	0.10 ± 0.05	0.57

* The data are based on 95th percentile values from the National Health and Nutrition Examination Survey (NHANES) conducted by the Centers for Disease Control and Prevention (CDC 2021). ** Blood arsenic (As) MAL values were obtained from the normal human levels reported in the Agency for Toxic Substances and Disease Registry (ATSDR 2007) guidance.

**Table 5:** Mean values of trace elements in serum and urine.

Trace Elements	Matrix	This Study (Mean ± SD)	Laboratory Reference
Cu (µg/dL)	Serum	75.51 ± 7.58	73–206
Cu (µg/g creat)	Urine	6.81 ± 2.18	7–72
Zn (µg/mL)	Serum	0.93 ± 0.09	0.6–1.06
Zn (µg/g creat)	Urine	320.1 ± 95.42	89–910
Se (µg/L)	Serum	66.76 ± 6.60	110–165
Se (µg/g creat)	Urine	12.96 ± 2.44	10–35
Mo (µg/L)	Serum	0.73 ± 0.30	0.3–2.0
Mo (µg/g creat)	Urine	44.79 ± 12.97	3.7–115
Co (µg/L)	Serum	0.25 ± 0.11	<1.0
Co (µg/g creat)	Urine	0.41 ± 0.28	<1.7

**Table 6:** Comparison of heavy metal and trace element concentrations with published data.

	PbB µg/dL	AsB µg/L	AsU µg/g Creat	HgB µg/L	CdB µg/L	CdU µg/g Creat	CrB µg/L	CuS µg/dL	SeS µg/L	ZnS µg/mL	MoS µg/L	CoS µg/L
This study	0.91	0.40	5.90	0.19	0.12	0.07	0.71	75.51	66.76	0.93	0.73	0.25
Goker & Aydın, 2000 Istanbul, Turkey [30]	5.5											
Ozden et al., 2004 Istanbul, Turkey [31]	8.4											
Vural&Guvendik,1983 Ankara, Turkey [32]	16.54											
Akinci et al., 2016 Ankara, Turkey >20 age [33]	2.51				1.27							
CHMS 2007–2009 20–39 age [34]	1.1			0.65	0.34	0.31						
CHMS 2018–2019 20–39 age [35]	0.71		5.0	0.74	0.24	0.15	0.23					
Goullé et al., 2005 >20 age, France [36]	2.6	5.0		3.0	0.31			107	119		0.95	0.49
Yildiz et al., 2023 Turkey [37]				0.96	0.23							
Akarsu, 2013 Tokat, Turkey [38]								137.78	73.35	1.09		
Koseoglu et al., 1996 Denizli, Turkey [39]								138.14				
Gunaldi et al., 2012 Istanbul, Turkey [40]									51.5			
Kilinc et al., 2010 K. Maraş, Turkey [41]									72.42	0.84		
Guvendik and Tumturk 1993 Ankara, Turkey [42]									63.66			

PbB: Lead Blood, AsB: Arsenic Blood, AsU: Arsenic Urine, HgB: Mercury Blood, CdB: Cadmium Blood, CdU: Cadmium Urine, CrB: Chromium Blood, CuS: Copper Serum, SeS: Selenium Serum, ZnS: Zinc Serum, MoS: Molybdenum Serum, and CoS: Cobalt Serum.

## Data Availability

All data and materials reported in the article are included in the manuscript and in the Supplementary File.

## Supplementary Materials

The following supporting information can be downloaded at: https://www.mdpi.com/article/10.3390/ijerph23020233/s1, Supplementary Material S1: Volunteer Questionnaire Form.
