# Supplementary material for: A Heavy Metal and Trace Element Biomonitoring Study in a Young Cohort (Aged 18–24) in Istanbul, Turkey
Source: Int J Environ Res Public Health. 2026 Feb 12;23(2):233. doi: 10.3390/ijerph23020233 (PMC12941121; doi:10.3390/ijerph23020233)
Supplement: Supplementary file 1 [file ijerph-23-00233-s001.zip › ijerph-3969671-supplementary.pdf]

VOLUNTEER QUESTIONNAIRE FORM

Date: __/__/___

Name Surname:

Sex:

Birth date: __/__/___

Lenght/Weight:

CRITERIA FOR INCLUSION

Do you feel good today? Yes ( ) No ( )

Are you between the ages of 18-24? Yes ( ) No ( )

EXCLUSION CRITERIA

Do you have a chronic disease? Yes ( ) No ( )

(Please specify if any)…

Do you have a drug that you use regularly? Yes ( ) No ( )

(Please specify if any)…

NUTRITION

1- Do you have a vitamin that you use regularly? Yes ( ) No ( )
(Please specify if any)…

2- Do you use fish oil supplements? Yes ( ) No ( )

What brand of water do you consume?

…

3- Do you use tools such as a dispenser in outdoor environments? Yes ( ) No ( )

4- Are you vegetarian? Yes ( ) No ( )

5- Do you follow a special diet? Yes ( ) No ( )

If yes, what kind of diet? ...

6- Do you consume alcohol? Yes ( ) No ( )

Did you drink alcohol in the 48 hours before sample collection? Yes ( ) No ( )

If your answer is yes, please indicate the type and amount of alcohol.

…

How often do you drink alcohol?

- 3 or more per week ( )
- 2 or less per week ( )
- 1 or 2 per month ( )
- 3 or 4 years ( )

What types of alcohol do you consume? (vine, beer, vodka, whiskey etc….)

…

How many years have you been using alcohol?

…

7- How often do you consume red meat?

Never ( )
2 or less per week ( )
3 or more per week ( )
Every day ( )

How often do you consume vegetables?
Never ( )
2 or less per week ( )
3 or more per week ( )
Every day ( )

8- Did you consume any seafood in the 3 days before sampling? Yes ( ) No ( )

If yes, what kind of seafood?

…

Where do you get the fish you consume?

From the markets ( ) From the restaurant ( )

From the fish market ( ) Others (please indicate)…

9- How many times do you consume seafood per week? (Fill in the table)

	**3-4 or more per week**	**1-2 times a week**	**1 time per month**	**Several times a year**	**1 or less per year**	**Never**
**Sushi**						
**Tuna**						
**Bonito fish**						
**Brean**						
**Anchovy**						
**Horse mackerel**						
**Clam**						
**Salmon**						
**Bass**						
**Blue fish**						
**Small blues fish**						
**Haddock**						
**Sardine**						
**Mackereş**						
**Shrimp**						
**Calamary**						
**Octopus**						
**Grey mullet**						
**Sole**						
**Small blues fish**						
**Red mullet fish**						
**Caviar**						
**Angler fish**						
**Coral**						
**Striped red mullet**						
**Crab**						
**Lobster**						
**Swordfish**						
**Trout**						
**Other (……………)**						
**Other (……………)**						
**Other (……………)**						

SOCIAL LIFE

10- Are you living with your family? Yes ( ) No ( )

In which district do you live in Istanbul?

…

Can you briefly describe where you live? (Highway side, central, rural, woodland etc.)

…

How many years have you been living in Istanbul?

…

11- How many hours do you spend in traffic during the day?

…

12- Do you smoke? Yes ( ) No ( )

If your answer is yes;

How many cigarettes do you smoke per day?

……………

How many years have you been smoking?

...

13- Do you smoke hookah? Yes ( ) No ( )

If yes, how often?

…

14- Do you use electronic cigarettes? Yes ( ) No ( )

15- Do you use anti-dandruff shampoo? Yes ( ) No ( )

16- Do you use a spot remover/skin lightening cream?? Yes ( ) No ( )

17- Do you have piercings on your body? Yes ( ) No ( )

18- Do you have a tattoo? Yes ( ) No ( )

MEDICAL BACKGROUND

19- Have you had any operation? Yes ( ) No ( )

If yes, what kind of operation?

…………………………………………………………………….

20- Do you have complaints such as constant headache, dizziness, weakness, distraction, chronic fatigue?

………

21- Do you have amalgam fillings? Yes ( ) No ( )

If yes, how many years?

…………………………………………….
